# Psychometric evaluation of the Questionnaire of Life Satisfaction (FLZ^M^) in a representative population sample

**DOI:** 10.1186/s40359-026-05021-3

**Published:** 2026-07-07

**Authors:** Tamara Schwinn, Judith Hirschmiller, Elmar Brähler, Jörg Wiltink, Rüdiger Zwerenz, Manfred E. Beutel, Mareike Ernst, Lina Krakau

**Affiliations:** 1 Department of Psychosomatic Medicine and Psychotherapy, University Medical Center of the Johannes Gutenberg-University Mainz, Untere Zahlbacher Str. 8, Mainz, 55131 Germany; 2 Clinic and Polyclinic for Psychosomatic Medicine and Psychotherapy, Rostock University Medical Center, Rostock, Germany; 3 University Cancer Center Mainz (UCT), University Medical Center of the Johannes Gutenberg-University Mainz, Mainz, Germany; 4 Department of Clinical Psychology, Psychotherapy and Psychoanalysis, Institute of Psychology, University of Klagenfurt, Klagenfurt, Austria; 5 Institute of Psychology, Child and Adolescent Clinical Psychology, University of Wuppertal, Wuppertal, Germany

**Keywords:** Validation, Psychometric evaluation, Life satisfaction, FLZ, Factor analysis

## Abstract

**Objective:**

Life satisfaction is a key construct within the salutogenetic framework of health and requires reliable assessment tools for research and clinical practice. This study evaluates the psychometric properties of the widely used Questionnaire of Life Satisfaction (FLZ^M^ for General Life Satisfaction).

**Methods:**

A total of *N* = 2,503 (53.10 % female) participants from a representative German population sample completed the questionnaire alongside measures of sociodemographic variables, depressive and anxiety symptoms, suicidal ideation, and mood. Item and scale characteristics, as well as internal consistency, were examined. The hypothesized two-factor structure (satisfaction and importance) was tested using a confirmatory factor analysis. Measurement invariance was assessed across gender, age groups, and the presence of depressive and anxiety symptoms. Group differences and criterion validity were analyzed.

**Results:**

The revised two-factor model, including correlated residuals for identical life domains, demonstrated improved fit compared to the proposed model. Internal consistency was good for both subscales (McDonald’s ω = .87 for the total scale). Measurement invariance was supported across all examined groups. As expected, life satisfaction was negatively correlated with depressive and anxiety symptoms, suicidal ideation, and negative mood states, and positively correlated with vigor.

**Conclusion:**

The FLZ^M^ for General Life Satisfaction showed solid psychometric properties and measurement invariance in a representative German sample. These findings support its validity as a reliable instrument for assessing life satisfaction, which can contribute to the improved implementation and development of targeted interventions at an individual level.

**Supplementary Information:**

The online version contains supplementary material available at 10.1186/s40359-026-05021-3.

## Background

According to the World Health Organization, health is not the absence of disease but the state of complete physical, mental and social well-being [1]. One important facet of well-being is *life satisfaction*, defined as „degree to which a person positively evaluates the overall quality of his/her life as a whole. In other words, how much the person likes the life he/she leads” (p. 6; [2]). This component of well-being is also conceptualized as a conscious cognitive evaluation of one’s life [3, 4]. Some work emphasizes its subjective nature and at times uses it synonymously with well-being [5].

Life satisfaction is of great scientific interest and has been examined in relation to a wide range of factors, including studies across the lifespan of individuals [6–8]. Research has shown that specific life satisfaction domains are associated with various outcomes such as psychological and physical health, health behaviors, and social factors [9]. Notably, lower life satisfaction is broadly associated with increased depression and anxiety [10–12], while suicidal behavior appears to be influenced by certain factors of life satisfaction [13], highlighting the clinical relevance of the construct.

From a salutogenetic perspective, for diagnostic purposes, and the selection, implementation or development of suitable interventions, a valid and reliable assessment of life satisfaction is essential. Daig et al. [14] presented the existing instruments: Life satisfaction Index A (LSI-A; [15]), Satisfaction with life scale (SWLS; [16]), Life Satisfaction Questionnaire (LSQ-32; [17]) as well as the Questionnaire of Life Satisfaction (FLZ; [18]), which can be supplemented by recent ones, such as Life Satisfaction Scale (LSS; [19], Bielefeld Life Satisfaction Scale (BIFL; [20]).

The FLZ is available in various modules, including the FLZ^M^ for General Life Satisfaction or Health [21]. Henrich and Herschbach [22] reported high internal consistency as well as content and convergent validity for these modules, and a highly negative correlation between the FLZ^M^ for General Life Satisfaction and the Beck Depression Inventory. An additional adaptation FLZ^M^ (KJ) includes a version for children and adolescents [23].

The FLZ^M^ for General Life Satisfaction is a German-language instrument that assesses life satisfaction across eight domains. It combines ratings of satisfaction with ratings of importance, allowing for the calculation of differentially weighted scores, which makes it a comparatively economical yet comprehensive assessment tool [22]. The FLZ^M^ has been re-standardized using large, representative samples [14] and widely applied, e.g., in older adults [24] or individuals with cancer [25]. It has previously been used for age- and gender-specific analyzes [26] and for cohort analysis [27].

Although several studies have reported normative data for the FLZ^M^ for General Life Satisfaction [14, 24], to our knowledge, no recent validation study based on a large representative population sample is available. However, such a validation is crucial to ensure the robustness and generalizability of the instrument for research and clinical use over time. The present study aims to address this gap by validating the FLZ^M^ for General Life Satisfaction in a representative German population sample. First, for this purpose, we examine the item and scale characteristics as well as reliability. Second, we test the hypothesized two-factor structure (satisfaction and importance) and investigate measurement invariance with respect to gender, age-groups, depressive and anxiety symptoms as well as suicidal ideation. Lastly, we compare groups in relation to sociodemographic variables and psychological factors and analyze the criterion validity, expecting negative associations with adverse psychological indicators.

## Methods

### Transparency and openness

This study’s design and its analysis were not preregistered. Due to ethical restrictions, the data cannot be made publicly available, but parts are available upon request. The request should be directed to the corresponding author.

### Participants and procedure

The representative sample of the German general population was recruited by the demographic research institute called USUMA GmbH (Berlin, Germany; Unabhängige Serviceeinrichtung für Umfragen, Methoden und Analysen; independent service for surveys, methods and analysis in market and social research).

Inclusion criteria were an age of at least 14 years (with informed custodian consent from parents or legal guardians for underaged participants) and sufficient knowledge of the German language. Sociodemographic data (including age, living situation, level of education, and income) were gathered via face-to-face interviews. Regarding gender, participants could choose male, female, or diverse (complying with Germany's civil status act (“Personenstandsgesetz”)). Subsequently, participants completed self-report questionnaires. The data collection followed a multistage sampling approach between May and June 2020, resulting in a representative sample in terms of age and gender. The participants gave their informed consent in accordance with the guidelines in the Declaration of Helsinki. For underaged participants informed custodian consent from parents or legal guardians was granted. All data were fully anonymized prior to data analysis. The study was approved by the Ethical Review Committee of the University of Leipzig (043/20-ek/).

### Instruments

*Life satisfaction* was assessed with the FLZ^M^ for General Life Satisfaction, which consists of eight items/life domains (friends/acquaintances, leisure time/hobbies, health, income/financial security, occupation/work, housing/living conditions, family life/children, and partner relationship/sexuality). For each domain, participants rate both the importance and their satisfaction on a Likert scale from 1 (Not important/Unsatisfied) to 5 (Extremely important/Very satisfied). Two scores can be calculated: an unweighted general life satisfaction score (mean value of the 8 items) and a weighted satisfaction score [14]. The FLZ^M^ was administered in its original German version. Both, the German version and an English translation can be found in the Supplementary Material.

*Anxiety symptoms* were assessed by the Generalized Anxiety Disorder scale (GAD-2; Kroenke et al. [28]) which is part of the Patient Health Questionnaire (PHQ-4; Löwe et al. [29], Wicke et al. [30]) and consists of two items “Feeling nervous, anxious or on the edge” and “Not being able to stop or control worrying”. The response scale ranged from 0 (Not at all) to 3 (Almost every day), also with a total score between 0 and 6. The cut-off score of ≥ 3 showed clinically relevant symptom burden [28]. In this study, Cronbach’s α was acceptable (α = 0.76).

*Depressive symptoms* were measured by the Patient Health Questionnaire (PHQ-2; Löwe et al. [31]), which is also part of the PHQ-4 and comprises two items asking for “Little interest or pleasure in doing things” and “Feeling down, depressed or hopeless”. Responses range from 0 (Not at all) to 3 (Almost every day) with a total score from 0 to 6. With a cut-off score of ≥ 3, the PHQ-2 showed good sensitivity and specificity for major depressive disorders [31]. In the present sample Cronbach’s α was acceptable (α = 0.71).

*Mood* was measured with the short version of the Profile of Mood Scales (POMS-16; Petrowski et al. [32]), a shortened scale of the long version (POMS-65; McNair et al. [33], McNair and Droppleman [34]). This questionnaire consists of the question “How have you felt during the last week, including today” with four items each on the subscales (Dejection, Vigor, Fatigue, and Anger) which can be answered on a scale from 0 (Not at all) to 6 (Very strong), giving a potential score of 24 on each subscale. No validated cut-off scores are available. Previous research by Schmalbach et al. [35] and the present study found high reliability for all subscales (α = 0.84–0.89).

*Suicidal Ideation* was assessed by item 9 of the Patient Health Questionnaire (PHQ-9; Kocalevent et al. [36], Lowe et al. [37], Martin et al. [38]), which asks about the presence of suicidal thoughts during the last week. Answers are given on a scale from 0 (Not at all) to 3 (Nearly every day). Responses were dichotomized into 0 = no suicidal ideation and ≥ 1 = suicidal ideation.

### Statistical procedures

All analyses were performed using R statistics version 4.4.2 using the packages: haven, car, psych, dplyr, lavaan, lavaanPlot, semTools, and rstatix. The code is available on the Open Science Framework (https://osf.io/e4bqg/).

*Item and scale characteristics* were examined via correlation between the subscales and the items with identical life domains of the subscales as well as psychometric item indices including missing rates for the items.

*Reliability* was assessed using internal consistency (Cronbach’s α for homogeneity, McDonald’s ω for not Tau-equivalent items, and ω_h_ for estimating how much variance is explained by a superordinate general factor [39, 40]). Additionally, average corrected item-total correlations examined item homogeneity and item-level reliability was calculated through hypothetical item omission to evaluate the contribution of each item to the overall reliability.

To analyze the proposed two-*factor structure*, a confirmatory factor analysis (CFA) was carried out with importance and satisfaction as separate factors. Given the ordinal structure, the robust weighted least squares estimator (WLSMV) was used [41]. To compare the model fit, a revised model with error covariances was specified between item pairs measuring the identical life domains to account for shared method variance. Model fit was evaluated in both models based on multiple fit indices and standardized parameter estimates: The cut-off for the Comparative Fit Index (CFI) is nearly ≥ 0.95 and for the Root Mean Square Error of Approximation (RMSEA) and the Standardized Root Mean Square Residual (SRMR) < 0.08 [42, 43].

The final model was visualized using a path diagram including standardized factor loadings and covariances.

To assess *measurement invariance,* sequential model estimations (with *configural* invariance testing whether the same factor structure holds across groups, *metric* invariance testing whether factor loadings are equal across groups, and *scalar* invariance testing whether item intercepts/thresholds are equal across groups [44–47]) were conducted for gender (men vs. women), age-groups (< 47 years vs. ≥ 47 years; age median of the sample), depressive (PHQ-2 sum score < 3 vs. ≥ 3) and anxiety symptoms (GAD-2 sum score < 3 vs. ≥ 3). As suicidal ideation was only measured by a single item and there is no cut-off for the subscales of the POMS-16, these items were not included. WLSMV was used and robust indices were reported for ordinal and non-normally distributed data [48, 49]. Nested models were compared using χ^2^ difference testing and changes in the CFI with a cut-off: ΔCFI ≤ -0.01 [50].

To *compare* importance and satisfaction between *groups,* a Wilcoxon-test was performed for gender, age-groups, depressive and anxiety symptoms, and suicidal ideation.

To test *criterion validity*, Pearson’s correlation between the subscales importance and satisfaction with depressive and anxiety symptoms, suicidal ideation, and the four subscales of the POMS-16 were analyzed.

## Results

### Sociodemographic sample description

The sample comprised 2,503 individuals (*M*(*SD*) = 45.99 (17.77), range:14–91 years). Of these, 1,173 identified as male, 1,329 as female, and 1 as diverse. In total, 41.95 % of the participants were married and 56.89 % had a high school education or higher. The majority of the sample held German nationality (95.88 %). While 59.13% of the sample were employed, 5.83 % were unemployed, and 20.18 % retired. Detailed sociodemographic and psychological information of the sample can be found in Table 1.

**Table 1:** Sociodemographic and psychological characteristics of the sample

			Total sample (*N* = 2,503)	Male subsample (*n* = 1,173)	Female subsample (*n* = 1,329)
			*N* (%)	*n* (%)	*n* (%)
Age *M* (*SD*)			45.99 (17.77)	45.28 (17.47)	46.64 (18.01)
Marital status					
Married, living with partner			981 (39.19)	484 (41.26)	497 (37.40)
Married, not living with partner			69 (2.76)	26 (2.22)	43 (3.24)
Single			992 (39.63)	511 (43.56)	480 (36.12)
Divorced			296 (11.83)	119 (10.14)	177 (13.32)
Widowed			149 (5.95)	25 (2.13)	124 (9.33)
Nationality					
German			2,400 (95.88)	1,119 (95.40)	1,280 (96.31)
Other			103 (4.11)	54 (4.60)	49 (3.69)
Education					
No qualification			60 (2.40)	31 (2.64)	29 (2.18)
Primary education			507 (20.26)	249 (21.23)	258 (19.41)
High school			1,012 (40.43)	458 (39.05)	554 (41.69)
Upper vocational education			78 (3.12)	40 (3.41)	38 (2.86)
Upper Secondary			399 (15.94)	160 (13.64)	238 (17.91)
College/university			364 (14.54)	194 (16.54)	170 (12.79)
Currently student			76 (3.04)	36 (3.07)	40 (3.01)
Employment					
Full-time			1,069 (42.71)	662 (56.44)	401 (30.17)
Part-time			411 (16.42)	67 (5.71)	277 (20.84)
In vocational or higher education			246 (9.83)	119 (10.14)	126 (9.48)
Unemployed			146 (5.83)	76 (6.48)	70 (5.27)
Retired			505 (20.18)	224 (19.10)	281 (21.14)
Other			95 (3.80)	14 (1.19)	81 (6.09)
Depressive symptoms (PHQ-2)					
Score < 3			954 (38.11)	497 (42.37)	457 (34.39)
Score ≥ 3			1,527 (61.01)	670 (57.12)	856 (64.41)
Anxiety symptoms (GAD-2)					
Score < 3			1,146 (45.33)	609 (51.92)	536 (40.33)
Score ≥ 3			1,339 (53.50)	556 (47.40)	783 (58.92)
Suicidal ideation (PHQ-9 item)					
No (Score = 0)			2,261 (90.61)	1,064 (90.71)	1,196 (89.99)
Yes (Score ≥ 1)			235 (9.39)	106 (9.04)	129 (9.71)
Mood (POMS)					
Dejection *M* (*SD*)			8.09 (4.76)	7.71 (4.41)	8.41 (5.03)
Vigor *M* (*SD*)			17.41 (4.92)	17.58 (4.96)	17.27 (4.96)
Fatigue *M* (*SD*)			11.79 (5.74)	11.24 (5.47)	12.27 (5.93)
Anger *M* (*SD*)			9.25 (5.23)	9.20 (5.13)	9.30 (5.31)

In certain cases, indicated numbers can be lower than the full sample size due to missing values. One participant identified as of diverse gender (complying with Germany's civil status act) and is included in the total score

*M* mean, *SD* standard deviation

### Item and scale characteristics

Correlations between items with identical life domains for importance and satisfaction were significant and positive in the low to moderate range. Smaller correlations were observed for items 3, 4, and 6 (see Table 2). The correlation between the two subscales of importance and satisfaction was *r* = 0.44 (*p* < 0.001).

**Table 2:** Correlation between importance and satisfaction ratings within the FLZ^M^ domains

Items	*r*	*p*
Friends/acquaintance	0.49	<.001
Leisure time/hobbies	0.42	<.001
Health	0.15	<.001
Income/financial security	0.13	<.001
Occupation/work	0.30	<.001
Housing/living conditions	0.25	<.001
Family life/children	0.47	<.001
Partner relationship/sexuality	0.45	<.001

*N* = 2,277

Complete data was available for 2,277 participants. The importance subscale showed negatively skewed distributions, indicating a tendency toward high ratings. Notably, item 3 demonstrated pronounced negative skewness and high kurtosis. Corrected item-total correlations ranged from *r*_it_^a^ = 0.39–0.55 with good to very good item homogeneity. Apart from item 5 with 2.24 %, the missing rate for the other items was below 1 %.

The distribution of the satisfaction subscale was also negatively skewed. Corrected item-total correlations ranged from *r*_it_^a^ = 0.48–0.61, reflecting a very good item selectivity. Missing rates were generally low (< 1.5 %), except for item 5 with over 5 % and item 8 with over 2 %. Item- and scale-level characteristics are summarized in Table 3.

**Table 3:** Item and scale characteristics of the Questionnaire of Life Satisfaction FLZ^M^

Subscale	Item	*M*	*SD*	Min; Max	Skewness	Kurtosis	*r*_it_^a^	missing rate (%)
Importance	1–8	31.79	4.57	8; 40	-0.67	1.05		
	1	3.84	0.89	1; 5	-0.48	-0.21	.39	0.48
	2	3.56	0.94	1; 5	-0.28	-0.40	.40	0.56
	3	4.58	0.68	1; 5	-1.85	4.15	.43	0.72
	4	4.20	0.81	1; 5	-0.83	0.40	.54	0.64
	5	3.76	1.07	1; 5	-0.88	0.37	.51	2.24
	6	4.03	0.80	1; 5	-0.58	0.18	.55	0.92
	7	4.06	1.07	1; 5	-1.10	0.57	.41	0.80
	8	3.74	1.13	1; 5	-0.80	-0.05	.47	0.92
Satisfaction	1–8	30.48	5.28	8; 40	-0.39	0.11		
	1	4.08	0.83	1; 5	-0.70	0.24	.50	0.64
	2	3.79	0.91	1; 5	-0.48	-0.04	.54	0.64
	3	3.80	1.00	1; 5	-0.63	-0.12	.50	0.80
	4	3.51	1.06	1; 5	-0.42	-0.40	.61	1.08
	5	3.56	1.08	1; 5	-0.54	-0.26	.60	5.51
	6	4.03	0.93	1; 5	-0.92	0.66	.52	0.56
	7	4.00	1.00	1; 5	-0.87	0.24	.50	1.40
	8	3.69	1.16	1; 5	-0.57	-0.58	.48	2.64

*N* = 2,277

1: friends/acquaintance, 2: leisure time/hobbies, 3: health, 4: income/financial security, 5: occupation/work, 6: housing/living conditions, 7: family life/children, 8: partner relationship/sexuality

*M* Mean, *SD* Standard Deviation, *Min* Minimum, *Max* Maximum, *r*_*it*_^*a*^ corrected item-total correlation

### Reliability

Internal consistency was good for both the importance subscale (α = 0.81; Confidence Interval (CI) [0.80;0.82]) and the satisfaction subscale (α = 0.81; CI[0.80;0.82]). The inter-item correlation was also good (importance: *r*_it_ = 0.29; satisfaction: *r*_it_ = 0.35).

For the total scale, ω was 0.87 and ω_h_ was 0.59 for both subscales, indicating the relevance of a general factor alongside meaningful subdimensions. Hypothetical item omission did not reduce reliability for any item, further supporting the internal consistency of the subscales.

### Factor structure

As the initial CFA showed an insufficient model fit (particularly regarding RMSEA), correlated residuals were assumed between pairs of items with identical content (of the identical life domains) to account for the method-related similarities (see Table 4).

**Table 4:** Two-factor model without and with correction

Model	CFI	TLI	RMSEA (90%-CI)	SRMR	χ^2^(*df*)	*p*
Two-factor model without correction	0.894	0.877	0.138 (0.135; 0.142)	0.101	5011.71 (103)	<.001
Two-factor model with correction	0.947	0.933	0.102 (0.099; 0.106)	0.075	2567.62 (95)	<.001

The factor model with correction assumes correlated residuals between pairs of items with identical life domains. Robust fit indices are reported

*CFI* Comparative Fit Index, *TLI* Tucker-Lewis Index, *RMSEA* Root Mean Square Error of Approximation, *90%-CI* 90%-Confidence Interval, *SRMR* Standardized Root Mean Square Residual, *χ*^*2*^ Chi-square test of model fit, *df* degrees of freedom

**Fig. 1:**
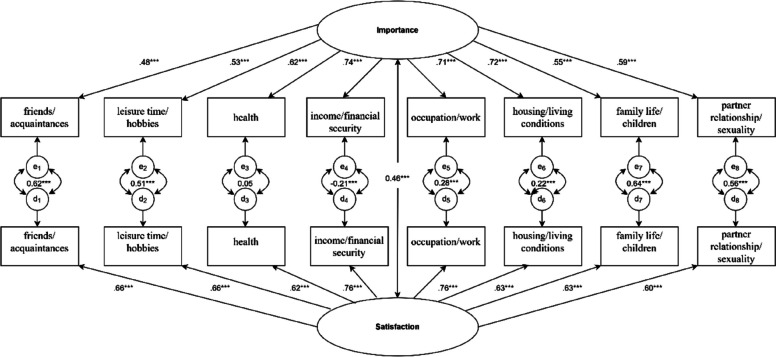
Confirmatory Factor analysis of the Questionnaire of Life Satisfaction FLZ^M^

The final model (two-factor model with correction) achieved acceptable standard fit indices (CFI = 0.947, TLI = 0.933, RMSEA = 0.102; SRMR = 0.075). However, the robust indices indicated a less favorable fit with a still relatively high RMSEA. The standardized factor loadings ranged from acceptable (e.g., “friends/acquaintance” for importance) to very good (e.g., “income/financial security” and “occupation/work” for satisfaction), suggesting varying strengths of the relationship between items and their respective latent factors. Eight correlated residuals were observed between the item pairs from the subscales, with a residual correlation between 0.05 (*p* > .05) for “health” and 0.64 (*p* < .001) for “family life/children”, which reflects method-related similarities. A negative residual correlation was found for “income/financial security” (*r* = -0.21, *p* < .001), pointing to diverging experiences across subscales. For detailed information, see Fig. 1.

### Measurement Invariance

Measurement invariance of the Questionnaire of Life Satisfaction FLZ^M^ was tested for gender, age-groups, and the presence of depressive and anxiety symptoms (see Table 5).

**Table 5:** Test of measurement invariance of the FLZ^M^

		χ^2^	*df*	*p*	CFI	TLI	RMSEA (90 %CI)	SRMR	Model Comparison	ΔCFI
Gender ^a^										
1. Configural invariance		2716.97	190	<.001	0.946	0.932	0.108 (0.105; 0.112)	0.078		
2. Metric invariance		2849.29	204	<.001	0.943	0.933	0.107 (0.103; 0.110)	0.079	1 vs. 2: Δχ^2^(14) = 70.37***	0.003
3. Scalar invariance		2792.53	236	<.001	0.945	0.944	0.098 (0.094; 0.101)	0.078	2 vs. 3: Δχ^2^(32) = -88.10	-0.002
Age-groups ^b^										
1. Configural invariance		2405.32	190	<.001	0.953	0.940	0.101 (0.098; 0.105)	0.075		
2. Metric invariance		2635.83	204	<.001	0.948	0.939	0.102 (0.099; 0.106)	0.079	1 vs. 2: Δχ^2^(14) = 117.23***	0.005
3. Scalar invariance		2498.78	236	<.001	0.952	0.951	0.092 (0.089; 0.095)	0.075	2 vs. 3: Δχ^2^(32) = 17.16	-0.004
Depressive symptoms ^c^										
1. Configural invariance		4229.28	152	<.001	0.871	0.845	0.154 (0.150; 0.158)	0.112		
2. Metric invariance		4254.82	164	<.001	0.870	0.856	0.148 (0.145; 0.152)	0.112	1 vs. 2: Δχ^2^(12) = 14.04	0.000
3. Scalar invariance		4270.56	192	<.001	0.871	0.877	0.137 (0.133; 0.141)	0.112	2 vs. 3: Δχ^2^(28) = 27.72	-0.000
Anxiety symptoms ^d^										
1. Configural invariance		4787.05	178	<.001	0.881	0.860	0.151 (0.147; 0.155)	0.109		
2. Metric invariance		4819.61	191	<.001	0.881	0.869	0.146 (0.143; 0.150)	0.109	1 vs. 2: Δχ^2^(13) = 17.33	0.001
3. Scalar invariance		4828.34	221	<.001	0.881	0.887	0.136 (0.132; 0.139)	0.109	2 vs. 3: Δχ^2^(30) = 17.31	-0.001

The measurement invariance was evaluated sequentially for configural, metric and scalar invariance. All reported fit indices are robust and estimated using WLSMV estimator

*χ*^*2*^ Chi-square test of model fit, *df* degrees of freedom, *CFI* Comparative Fit Index, *TLI* Tucker-Lewis Index, *RMSEA* Root Mean Square Error of Approximation, *90 %-CI* 90 %-Confidence Interval, *SRMR* Standardized Root Mean Square Residual, *Δ* difference between nested models; ***p <.001

^a^Comparison of male and female participants; diverse could not be included in this analysis due to small sample size

^b^Comparison of persons < 47 and ≥ 47 years

^c^Comparison of persons with < 3 and ≤ 3 PHQ-2 sum scores

^d^Comparison of persons with < 3 and ≤ 3 GAD-2 sum scores

For *gender*, the measurement invariance models showed acceptable fit indices, although the RMSEA values exceeded the recommended threshold. The Δχ^2^-test for metric invariance was significant, whereas the change in CFI was below the recommended cut-off. Likewise, scalar invariance was supported by a negligible change in CFI. Overall, a configural, metric, and scalar invariance can be assumed.

*Age groups* showed a similar pattern, with the measurement invariances showing acceptable fit indices, except RMSEA values. Again, the Δχ^2^-tests and the CFI suggests configural, metric, and scalar invariance.

For *depressive symptoms*, items 3 and 4 of the importance subscale and for *anxiety symptoms* item 3 of the importance subscale had to be excluded due to a lack of response variability on these certain items (missing value 1). Although for depressive and anxiety symptoms, the measurement invariance models demonstrated a weak model fit, the model comparison indices indicate configural, metric, and scalar invariance.

### Group comparison

No significant differences were found between *gender* for either subscale.

A*ge groups* differed in importance with a small effect size (*W* = 874749, *p* < .001, *r* = 0.18) and, to a lesser extent, in satisfaction (*W* = 692434, *p* = .012, *r* = 0.033), with younger participants reporting higher scores in both subscales.

For *depressive symptoms,* there was a significant difference, with people with higher depression scores rating both importance (*W* = 790477, *p* < .001, *r* = 0.14) and satisfaction (*W* = 901376, *p* < .001, *r* = 0.38) lower, with a small to medium effect for the differences.

Participants with high *anxiety symptom* scores showed lower rates for importance (*W* = 789419, *p* < .001, *r* = 0.10) and especially satisfaction (*W* = 905368, *p* < .001, *r* = 0.33), with a small to medium effect.

Participants with *suicidal ideation* reported lower importance (*W* = 294364, *p* < .001, *r* = 0.11) and satisfaction (*W* = 314291, *p* < .001, *r* = 0.20) than participants without, with a small effect for both.

### Criterion validity

As shown in Table 6, both subscales correlated significantly and negatively with depressive symptoms, anxiety symptoms, and suicidal ideation. For the POMS-16 subscales, Dejection, Anger, and Fatigue were negatively associated with both importance and satisfaction, while Vigor showed a significant positive correlation with both subscales.

**Table 6:** Correlation (Pearson’s *r*) of subscales of the FLZ^M^ Life satisfaction with psychological constructs

	Depressive symptoms	Anxiety symptoms	Suicidal ideation	Mood: Dejection	Mood: Vigor	Mood: Fatigue	Mood: Anger
Importance	-0.13***	-0.06**	-0.13***	-0.14***	0.26***	-0.08***	-0.07***
Satisfaction	-0.44***	-0.37***	-0.19***	−0.47***	0.43***	-0.37***	-0.35***

**p <.01; ***p<.001

## Discussion

The present study aimed to evaluate the psychometric properties of the FLZ^M^ for General Life Satisfaction in a large, representative German population sample. Specifically, we examined item and scale characteristics, reliability, factorial validity, and measurement invariance across demographic and clinical subgroups, as well as associations with indicators of mental health. Overall, the results support the FLZ^M^ as a reliable and valid instrument for assessing life satisfaction, although several limitations warrant caution in interpreting the findings.

Both of its subscales, importance and satisfaction, showed good internal consistency, and item-total correlations were satisfactory across domains which supports findings from norming studies finding internal consistency of α = 0.82 [22, 27] and similar to other scales such the LSI-A [51] or the SWLS [27, 52]. Moreover, the McDonald’ ω also replicates prior findings with ω = 0.78 to 0.82 across samples from 1991 and 2020 [22, 27]. Criterion validity was demonstrated by the expected negative associations with depressive and anxiety symptoms, suicidal ideation, and negative mood states, alongside a positive association with vigor. This is consistent with previous findings reporting high negative correlations between depression and anxiety with the FLZ^M^ [22]. The small, but statistically significant correlations between the Importance subscale and both anxiety symptoms and the Mood subscale Fatigue should be interpreted with caution. As the sample size increases, even very small effects are more likely to reach statistical significance [53]. Given that these associations represent small Pearson correlations, it is important to note that statistical significance does not necessarily imply clinical or practical significance, i.e., meaningful relevance in real-world contexts [54].

Group comparisons revealed meaningful differences: individuals with depressive or anxiety symptoms, as well as those reporting suicidal ideation, consistently showed lower satisfaction scores, underlining the clinical relevance of the instrument. Age differences also emerged, with younger participants reporting higher importance and, to a lesser extent, satisfaction, whereas no differences between women and men were observed.

The CFA provided partial support for the hypothesized two-factor model. Although the revised model, including correlated residuals, yielded improved fit indices, the overall model fit remained below optimal thresholds, suggesting that the assumed structure only partially reflects the empirical data. Research has shown that RMSEA may overreject models under certain conditions [55].

Measurement invariance analyses largely supported configural, metric, and scalar invariance (mostly interpreting ΔCFI as recommended for large samples [50]) across gender, age-groups, and depressive and anxiety symptom groups. However, invariance results should be interpreted cautiously, as several items had to be excluded in the depressive and anxiety symptom subgroups due to limited response variability. This restriction may have introduced distortions and limits the validity of the invariance results.

In interpreting these findings, several limitations and critical aspects should be considered. First, the relatively high proportion of missing values in “occupation/work” for both the importance and satisfaction subscales reflect participants’ current unemployment status or retirement, which made the item less applicable (of the 56 participants who did not answer this item for importance, 43 were retired, 2 were unemployed, and 2 were still in school. Of the 137 participants who did not answer this item for satisfaction, 94 were retired, 11 unemployed, and 21 still in school; the remaining were employed or did not provide information). Similarly, the elevated missing rate for “partnership/sexuality” in the satisfaction subscale could be explained by participants without a current partnership choosing not to respond (out of the 66 participants who did not answer this item for satisfaction 54 were not in a relationship). Second, the correlations between importance and satisfaction were particularly low in the domains of health and income/financial security. This may be due to the fact that these domains often show the greatest discrepancy between importance (typically rated as high) and satisfaction (potentially low in cases of illness, low income, or debt), and they may also be less subject to personal control than other domains. Another study also revealed the greatest differences between Western and Eastern Germany in terms of financial satisfaction [27].

Regarding the factor structure, although the revised two-factor model showed an improved fit, overall fit indices remained less than optimal. In particular, the negative residual correlation observed for the financial domain suggests that importance and satisfaction items share additional variance not fully captured by the latent factors, warranting further investigation.

### Constraints on generality

The present findings of this study support the validity and psychometric robustness of the FLZ^M^ in a representative German population sample (in terms of age and gender). Given that, the results can be considered generalizable. However, only one participant identified as gender diverse. Moreover, no gender-sensitive distinction was made regarding trans- or cis-gender classification. Given the potential impact of minority stress processes on these populations, as described by the minority stress model [56, 57], further research on life satisfaction in these groups, as well as the development of targeted recruitment strategies to better reach them, would be particularly valuable.

## Conclusion

Future research should aim to replicate and extend the present analyses using independent samples and alternative factor structures. Moreover, longitudinal datasets that include objective measurements would provide complementary evidence and further strengthen and clarify the findings. In addition, comparisons with newly developed instruments, such as the BIFL [20], may provide further insights into the relative strengths and limitations of the FLZ^M^. Finally, more studies are needed to explore the potential applications of the FLZ^M^ in clinical practice, for example, to guide individualized diagnostic assessments and inform counselling and intervention planning in clinical contexts.

## Supplementary Information

Supplementary Material 1. 

## Data Availability

The authors confirm that, for approved reasons, some access restrictions apply to the data underlying the findings. Due to ethical restrictions, the data cannot be made publicly available, but parts are available upon request. The request should be directed to the corresponding author. The FLZ^M^ was administered in its original German version. Both, the German version and an English translation can be found in the Supplemental Material. The code can be found in the Open Science Framework (https://osf.io/e4bqg/).
