# Supplementary material for: Psychometric evaluation of the Questionnaire of Life Satisfaction (FLZM) in a representative population sample
Source: BMC Psychol. 2026 Jul 7;14:995. doi: 10.1186/s40359-026-05021-3 (PMC13339703; doi:10.1186/s40359-026-05021-3)
Supplement: Supplementary file 1 — Supplementary Material 1. [file 40359_2026_5021_MOESM1_ESM.docx]

German version of the FLZ^M^

Bei den folgenden Fragen geht es darum, wie zufrieden Sie mit Ihrem Leben und mit einzelnen Aspekten Ihres Lebens sind. Außerdem sollen Sie angeben, wie wichtig einzelne Lebensbereiche (z. B. Beruf oder Freizeit) für Ihre Zufriedenheit und Ihr Wohlbefinden sind.

Bitte kreuzen Sie zunächst an, wie wichtig jeder einzelne Lebensbereich für Ihre Zufriedenheit insgesamt ist. Bevor Sie beginnen, schauen Sie bitte erst alle Bereiche an.

Bitte kreuzen Sie in jeder Zeile an, was für Sie zutrifft.

| Wie **wichtig** ist (sind) für Sie… | Nicht wichtig | Etwas wichtig | Ziemlich wichtig | Sehr wichtig | Extrem wichtig |
| --- | --- | --- | --- | --- | --- |
| Freunde / Bekannte | O | O | O | O | O |
| Freizeitgestaltung / Hobbys | O | O | O | O | O |
| Gesundheit | O | O | O | O | O |
| Einkommen / Finanzielle Sicherheit | O | O | O | O | O |
| Beruf / Arbeit | O | O | O | O | O |
| Wohnsituation | O | O | O | O | O |
| Familienleben / Kinder | O | O | O | O | O |
| Partnerschaft / Sexualität | O | O | O | O | O |

Bitte kreuzen Sie nun an, wie zufrieden Sie in den einzelnen Lebensbereichen sind.

| Wie **zufrieden** sind Sie mit … | Unzufrieden | Eher unzufrieden | Eher zufrieden | Ziemlich zufrieden | Sehr zufrieden |
| --- | --- | --- | --- | --- | --- |
| Freunde / Bekannte | O | O | O | O | O |
| Freizeitgestaltung / Hobbys | O | O | O | O | O |
| Gesundheit | O | O | O | O | O |
| Einkommen / Finanzielle Sicherheit | O | O | O | O | O |
| Beruf / Arbeit | O | O | O | O | O |
| Wohnsituation | O | O | O | O | O |
| Familienleben / Kinder | O | O | O | O | O |
| Partnerschaft / Sexualität | O | O | O | O | O |

English version of the FLZ^M^

The following questions concern how satisfied you are with your life and with individual aspects of your life. You are also asked to indicate how important different areas of life (e.g., work or leisure) are for your satisfaction and well-being.

Please first check how important each area of life is for your overall satisfaction. Before you begin, please look through all the areas.

Please check in each row what applies to you.

| How **important** is (are) … | not important | rather not important | rather important | very important | extremely important |
| --- | --- | --- | --- | --- | --- |
| friends/acquaintances | O | O | O | O | O |
| leisure time/hobbies | O | O | O | O | O |
| health | O | O | O | O | O |
| income/financial security | O | O | O | O | O |
| occupation/work | O | O | O | O | O |
| housing/living conditions | O | O | O | O | O |
| family life/children | O | O | O | O | O |
| partner relationship/sexuality | O | O | O | O | O |

Please now check how satisfied you are with each area of your life.

| How **satisfied** are you … | unsatisfied | rather unsatisfied | rather satisfied | very satisfied | extremely satisfied |
| --- | --- | --- | --- | --- | --- |
| friends/acquaintances | O | O | O | O | O |
| leisure time/hobbies | O | O | O | O | O |
| health | O | O | O | O | O |
| income/financial security | O | O | O | O | O |
| occupation/work | O | O | O | O | O |
| housing/living conditions | O | O | O | O | O |
| family life/children | O | O | O | O | O |
| partner relationship/sexuality | O | O | O | O | O |

*Note.* The FLZ^M^ was administered in its original German version. The English translation is partly based on information from other work [1-3].

References

[1] G. Henrich and P. Herschbach, "Questions on Life Satisfaction (FLZM): a short questionnaire for assessing subjective quality of life," *European Journal of Psychological Assessment,* vol. 16, no. 3, p. 150, 2000.

[2] K. Leuteritz, M. Friedrich, A. Sender, E. Nowe, Y. Stoebel‐Richter, and K. Geue, "Life satisfaction in young adults with cancer and the role of sociodemographic, medical, and psychosocial factors: results of a longitudinal study," *Cancer,* vol. 124, no. 22, pp. 4374-4382, 2018.

[3] A. Hinz, M. Zenger, B. Schmalbach, E. Brahler, D. Hofmeister, and K. Petrowski, "Quality of Life Domains in Breast Cancer Survivors: The Relationship Between Importance and Satisfaction Ratings," *Front Psychol,* vol. 13, p. 923537, 2022, doi: 10.3389/fpsyg.2022.923537.
